# Effects of Cadmium Stress on Carbon Sequestration and Oxygen Release Characteristics in A Landscaping Hyperaccumulator—*Lonicera japonica* Thunb.

**DOI:** 10.3390/plants12142689

**Published:** 2023-07-19

**Authors:** Zhouli Liu, Jing An, Qingxuan Lu, Chuanjia Yang, Yitao Mu, Jianbing Wei, Yongxia Hou, Xiangyu Meng, Zhuo Zhao, Maosen Lin

**Affiliations:** 1 College of Life Science and Engineering, Shenyang University, Shenyang 110044, China; zlliu@syu.edu.cn (Z.L.); lqx0812@syu.edu.cn (Q.L.); oliver1208@sina.com (J.W.); houyongxia@syu.edu.cn (Y.H.); mengxy559@syu.edu.cn (X.M.); zhaozhuo_86@163.com (Z.Z.); 2 Northeast Geological S & T Innovation Center of China Geological Survey, Shenyang 110000, China; 3 Key Laboratory of Pollution Ecology and Environmental Engineering, Institute of Applied Ecology, Chinese Academy of Sciences, Shenyang 110016, China; 4 Department of General Surgery, Shengjing Hospital, China Medical University, Shenyang 110004, China; cjyang@cmu.edu.cn; 5 College of Municipal and Environmental Engineering, Shenyang Urban Construction University, Shenyang 110167, China; faximcql@163.com; 6 College of Water Conservancy, Shenyang Agricultural University, Shenyang 110161, China; fangbing_219900@163.com

**Keywords:** heavy metal, *Lonicera japonica* Thunb., carbon sequestration, oxygen release

## Abstract

The carbon sequestration and oxygen release of landscape plants are dominant ecological service functions, which can play an important role in reducing greenhouse gases, improving the urban heat island effect and achieving carbon peaking and carbon neutrality. In the present study, we are choosing *Lonicera japonica* Thunb. as a model plant to show the effects of Cd stress on growth, photosynthesis, carbon sequestration and oxygen release characteristics. Under 5 mg kg^−1^ of Cd treatment, the dry weight of roots and shoots biomass and the net photosynthetic rate (P_N_) in *L. japonica* had a significant increase, and with the increase in Cd treatment concentration, the dry weight of roots and shoots biomass and P_N_ in the plant began to decrease. When the Cd treatment concentration was up to 125 mg kg^−1^, the dry weight of root and shoots biomass and P_N_ in the plant decreased by 5.29%, 1.94% and 2.06%, and they had no significant decrease compared with the control, indicating that the plant still had a good ability for growth and photoenergy utilization even under high concentrations of Cd stress. The carbon sequestration and oxygen release functions in terms of diurnal assimilation amounts (*P*), carbon sequestration per unit leaf area (W_CO_2__), oxygen release per unit leaf area (W_O_2__), carbon sequestration per unit land area (P_CO_2__) and oxygen release per unit land area (P_O_2__) in *L. japonica* had a similar change trend with the photosynthesis responses under different concentrations of Cd treatments, which indicated that *L. japonica* as a landscaping Cd-hyperaccumulator, has a good ability for carbon sequestration and oxygen release even under high concentrations of Cd stress. The present study will provide a useful guideline for effectively developing the ecological service functions of landscaping hyperaccumulators under urban Cd-contaminated environment.

## 1. Introduction

Environmental pollution, including heavy metals and greenhouse gas, is gradually intensified as population growth and industrial agglomeration continue to expand [1,2,3]. A long-term excess of heavy metals such as cadmium (Cd) and greenhouse gases such as carbon dioxide (CO_2_) has been released into the environment, which has seriously threatened human health and survival as well as urban sustainable development [4,5,6,7]. This has also virtually increased the pressure on the developing countries in the world to practice carbon peaking and carbon neutrality. Therefore, these environmental issues have attracted widespread attention from countries around the world [8,9,10]. Landscape plants are an important foundation for urban greening construction and play a significant role in improving the urban environment. Several studies reported that hazardous heavy metals in urban soil can be adsorbed and removed by ornamental plants [11,12,13,14,15,16,17,18,19,20]. It was shown that the carbon sequestration and oxygen release of landscape plants are dominant ecological service functions, which play an irreplaceable role in reducing concentrations of atmospheric CO_2_ through the conversion process of inorganic carbon to organic compounds using plant organs [21,22]. 

It is known that among heavy metals, Cd is one of the most hazardous pollutants because of its high water solubility and strong cancerogenic effects, which can not only lead to leaf chlorosis, growth inhibition and photosynthetic rates decrease but can also be easily transferred into the food chain and threaten human health [23,24,25]. Currently, most research focuses on the accumulation ability, photosynthetic capacity and antioxidant enzyme activity of crops and mining plants under Cd stress [26,27,28,29,30,31,32,33,34,35]. However, little information is available on the carbon fixation and oxygen release characteristics in landscape plants, especially ornamental woody plants. 

*Lonicera japonica* Thunb., as a popular ornamental woody plant, has become established in temperate and tropical regions worldwide in the past 150 years [36]. It is widely used in urban greening because of its high biomass, easy cultivation and strong resistance to environmental stress, as well as its development in the pharmaceutical field [37]. In our previous studies, it was found that *L. japonica* is a new woody Cd-hyperaccumulator and had a good phytoremediation ability for Cd-contaminated soil [11,38,39,40,41,42]. As a typical landscaping plant, *L. japonica* has a good function of carbon sequestration and oxygen release. Therefore, in the present study, we are choosing *L. japonica* as a model plant to show the response characteristics of growth, photosynthesis, carbon sequestration and oxygen release functions in the plant under different concentrations of Cd stress. It will be helpful to effectively develop the ecological service functions of landscape plants under the urban Cd-contaminated environment and expand the application value of hyperaccumulators. 

## 2. Materials and Methods

### 2.1. Plant Culture and Cd Treatments

The soil in the experiment was collected from the topsoil at Shenyang Agricultural University (41°44′ N and 123°27′ E, 44.7 m a.s.l.). The type of the tested soil is meadow, and its physical and chemical properties include a pH of 7.26 ± 0.03, organic matter (OM) content of 20.53 ± 0.05 g kg^−1^ and cation exchange capacity (CEC) of 18.95 ± 0.06 cmol kg^−1^. The mean concentration of extractable Cd in the tested soil was 0.15 ± 0.01. 

The air-dried tested soil was sieved through a 3 mm mesh sieve and placed into a plastic pot that measured 30 cm across. Then, the tested soil in each plastic pot was mixed uniformly with different concentrations of a Cd^2+^ solution derived from CdCl_2_·2.5H_2_O (Kermel Chemical Reagent Co., Ltd., Shanghai, China, >99%). Three 6-month-old seedlings with consistent growth were planted in each plastic pot. These plants were cultivated in a greenhouse with 75% relative humidity, 23 ± 2 °C temperature and 800–1000 μmol m^−2^ s^−1^ PPFD (16/8 h light/dark). The experiment set had four Cd treatment levels, which contained 0 (CK), 5, 25 and 125 mg kg^−1^, respectively. Each Cd treatment experiment consisted of four replicates. The soil water content was measured in each pot using time domain reflectometry. After a 90 d Cd treatment, the plants were harvested for analysis.

### 2.2. Detection of Plant Biomass and Photosynthetic Parameters

The harvested plants were washed with tap water and then washed with deionized water. The plants were separated into leaves and roots. These plant tissues were dried at 105 °C for 20 min, then at 70 °C until the weight was constant. Subsequently, the dry weight (g) of roots and shoots biomass was obtained.

The net photosynthetic rate (P_N_) of the plant is one of the most important photosynthetic parameters, which was determined using a portable LI-6400 photosynthesis system (Lincoln, NE, USA) under different Cd stress. During different Cd treatments, the parameters (light level, CO_2_ concentration and leaf temperature) inside the leaf chamber were maintained stable at 1000 μmol m^−2^ s^−1^ PPFD, 25 ± 0.3 °C and 380 ± 5 μmol CO_2_ mol^−1^. 

### 2.3. Assays of Carbon Sequestration and Oxygen Release Functions

The determination of carbon sequestration and oxygen release functions were referenced in the relative study [34]. The carbon sequestration and oxygen release values of the plant were derived from diurnal assimilation amounts (*P*), which could be represented with the following Equation (1):

(1) $$P=\sum_{i=1}^{j}\left[(p_{i+1}+p_{i})÷2\times (t_{i+1}-t_{i})\times 3600÷1000\right]$$

where *P* is diurnal assimilation amounts (mmol m^−2^ s^−1^), *p_i_* is instantaneous photosynthetic rate at the initial measurement point (μmol m^−2^ s^−1^), *p_i+_*_1_ is instantaneous photosynthetic rate of the next measurement point (μmol m^−2^ s^−1^), *t_i_* is instantaneous time of initial measurement point (h), *t_i_* is instantaneous time of the next measurement point (h), *j* is test times, 3600 refers to 3600 s per hour and 1000 refers to 1000 μmol per mmol.

Carbon sequestration per unit leaf area (W_CO_2__) and oxygen release per unit leaf area (W_O_2__) of the plant could be represented with the following Equations (2) and (3):

W_CO_2__ = *P* × 44/1000 (2)

W_O_2__ = *P* × 32/1000 (3)

where W_CO_2__ is carbon sequestration per unit leaf area (g m^−2^ d^−1^), 44 is the molar mass of CO_2_; W_O_2__ is oxygen release per unit leaf area (g m^−2^ d^−1^) and 32 is the molar mass of O_2_.

Carbon sequestration per unit land area (P_CO_2__) and oxygen release per unit land area (P_O_2__) of the plant could be represented with the following Equations (4) and (5):

P_CO_2__ = W_CO_2__ × E (4)

P_O_2__ = W_O_2__ × E (5)

where P_CO_2__ is carbon sequestration per unit land area (g m^−2^ d^−1^), P_O_2__ is oxygen release per unit land area (g m^−2^ d^−1^) and E is leaf area index.

### 2.4. Statistical Analyses

The data analyses in the study were performed as the means ± SD. The statistical analysis of the data was applied using SPSS 22.0 and Microsoft Office Excel 2020. The level of significant difference was presented at *p* < 0.05 or *p* < 0.01.

## 3. Results and Discussion

### 3.1. Effects of Different Cd Treatments on Plant Growth

**Figure 1:**
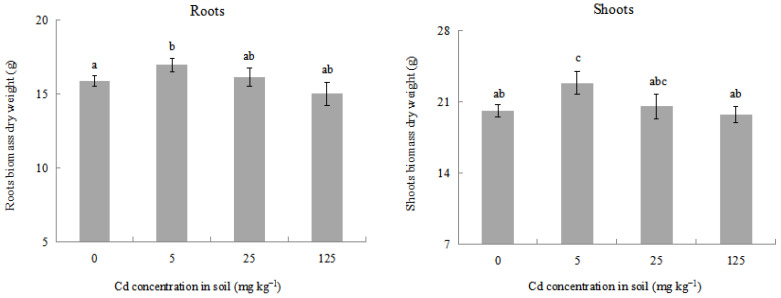
Effects of different Cd treatments on dry weight of roots and shoots biomass in *L*. *japonica*. Values represent mean ± SD. Different letters represent significant differences at the 5% level according to the LSD test.

It is reported that the changes in plant biomass are an important susceptible parameter when the plants are subjected to heavy metals or other environmental stress [43,44,45]. After a 90 d Cd treatment, the growth responses in terms of the dry weight of roots and shoots biomass in *L. japonica* under Cd different treatments are shown in Figure 1. The dry weight of roots and shoots biomass represented similar change trends with the increase in Cd treatment concentration. Under 5 mg kg^−1^ of Cd treatment, the dry weight of roots and shoots biomass showed a significant increase compared with the control, which indicated that a low concentration of Cd treatment could stimulate plant growth. A similar phenomenon has been found in several studies when plants were exposed to a low Cd concentration in the medium, which is described as hormesis, and potentially displayed the “overcompensation” behavior when the homeostasis of an organism was disrupted [46,47,48,49,50,51]. The present result is consistent with our previous studies. With the increase in Cd treatment concentration, the dry weight of roots and shoots biomass in *L. japonica* all began to decrease. When Cd treatment concentration was up to 125 mg kg^−1^, the dry weight of roots and shoots biomass in *L. japonica* decreased, respectively, by 5.29% and 1.94%. Its differential change may be due to the first direct contact of plant roots with Cd^2+^ in soil; however, the dry weight of roots and shoots biomass had no significant decrease compared with the control, which is in agreement with no obvious injury symptoms in *L. japonica*.

### 3.2. Effect of Different Cd Treatments on Photosynthetic Parameters

**Figure 2:**
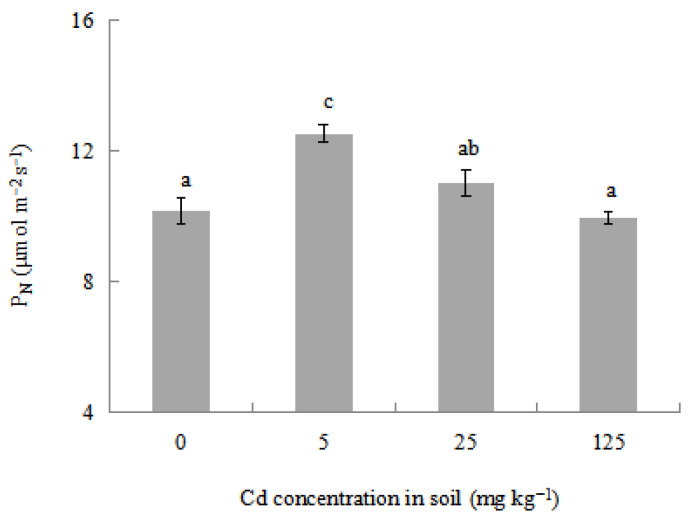
Effects of different Cd treatments on net photosynthesis rate (P_N_) in *L*. *japonica*. Values represent mean ± SD. Different letters represent significant differences at the 5% level according to the LSD test.

As already known, photosynthesis is a highly susceptible indicator of Cd stress [52,53]. The net photosynthesis rate (P_N_) is often used to represent the potential of plant photoenergy utilization [54]. The effects of different Cd treatments on P_N_ in *L. japonica* are shown in Figure 2. Several studies reported that Cd^2+^ had negative effects on P_N_ in some plants [53,55,56,57]. However, it was shown that photosynthesis in *Brassica juncea* had no obvious change when the plant was exposed to 25 mmol L^−1^ of Cd^2+^ stress [58]. Mustard (*Brassica juncea* L. Czern and Coss.) cvs. Varuna also showed an increased P_N_ when the plant was subjected to 200 mg kg^−1^ of Cd in soil [59]. In the present study, P_N_ in *L. japonica* has a significant increase of 23.21% compared with the control when the plant was exposed to 5 mg kg^−1^ of Cd treatment, which may result from a priming effect by hormesis. The significant increase in P_N_ may demonstrate improved growth in a short period of time, which corresponds to the growth responses in terms of the dry weight of roots and shoots biomass in *L. japonica* under 5 mg kg^−1^ of Cd treatment. With the increase in Cd treatment concentration, P_N_ in *L. japonica* began to decrease. When Cd treatment concentration was up to 125 mg kg^−1^, P_N_ in *L. japonica* decreased by 2.06% and had no significant decrease compared with the control, indicating that the plant still had a good ability of photoenergy utilization even under high concentrations of Cd stress. A similar phenomenon was also shown in photosynthesis responses in rice and maize under Cd stress [60].

### 3.3. Effect of Different Cd Treatments on Carbon Sequestration and Oxygen Release Functions

Carbon sequestration and oxygen release functions are regarded as critical parameters to assess photosynthetic ability, which can provide a useful guideline for the ecological service function of landscape plants [61,62]. The carbon sequestration and oxygen release functions of *L. japonica* exposed to different Cd treatments are shown in Table 1. In the present study, diurnal assimilation amounts (*P*), carbon sequestration per unit leaf area (W_CO_2__), oxygen release per unit leaf area (W_O_2__), carbon sequestration per unit land area (P_CO_2__) and oxygen release per unit land area (P_O_2__) of *L. japonica* has significant increases, going up to 271.63 ± 6.92 mmol m^−2^ s^−1^, 12.30 ± 0.32 g m^−2^ d^−1^, 8.95 ± 0.10 g m^−2^ d^−1^, 25.59 ± 0.65 g m^−2^ d^−1^ and 18.61 ± 0.37 g m^−2^ d^−1^, respectively, when the plant is exposed to 5 mg kg^−1^ Cd treatment. The significant increase in carbon sequestration and oxygen release values of *L. japonica* may stem from the effect of hormesis, which is highly relevant to the value of P_N_. The present study is in accordance with the report by [63,64], which proposed that landscape plants with functional carbon sequestration and oxygen release can exchange more CO_2_ and O_2_ with the external environment, then transform more solar energy into organic matter stored in plants. It is known that all the principal biotic components of carbon sequestration and oxygen release stem from the photosynthetic process of plants [21,65]. With the increase in Cd treatment concentration, *P*, W_CO_2__, W_O_2__, P_CO_2__ and P_O_2__ in *L. japonica* began to decrease; however, when Cd treatment concentration was up to 125 mg kg^−1^, *P*, W_CO_2__, W_O_2__, P_CO_2__ and P_O_2__ in *L. japonica* had no significant decrease compared with the control, which indicated a good photosynthetic ability of the plant even under high concentrations of Cd treatment.

## 4. Conclusions

In our previous studies, it was shown that *Lonicera japonica* Thunb. is not only a popular landscape plant to decorate the environment, but it also has a good remediation ability to remove toxic heavy metals. In the present study, the carbon sequestration and oxygen release functions in terms of *P*, W_CO_2__, W_O_2__, P_CO_2__ and P_O_2__ in *L. japonica* have a similar change trend with the photosynthesis responses under different concentrations of Cd treatments, which may result from all the principal biotic components of carbon sequestration and oxygen release provided by the photosynthetic process of plants. As a new woody Cd-hyperaccumulator, *L. japonica* is observed to have a good ability for carbon sequestration and oxygen release even under high concentrations of Cd stress. The present study will provide a useful guideline for the ecological service function of *L. japonica* in urban landscaping construction, showing that the rational application of the plant can not only phytoremediate urban Cd-contaminated soil, but it can also play an important role in reducing the greenhouse gases, improving the urban heat island effect and achieving carbon peaking and carbon neutrality. In future work, it is necessary to delve into the correlation between carbon sequestration capacity and physiological mechanisms.

## Figures and Tables

**Table 1:** The carbon sequestration and oxygen release functions of *L. japonica*.

Different Cd Treatments (mg kg^−1^)	Diurnal Assimilation Amounts (*P*, mmol m^−2^ s^−1^)	Carbon Sequestration per Unit Leaf Area (W_CO_2__, g m^−2^ d^−1^)	Oxygen Release per Unit Leaf Area (W_O_2__, g m^−2^ d^−1^)	Carbon Sequestration per Unit Land Area (P_CO_2__, g m^−2^ d^−1^)	Oxygen Release per Unit Land Area (P_O_2__, g m^−2^ d^−1^)
0	232.58 ± 4.86	10.23 ± 0.19	7.44 ± 0.12	16.89 ± 0.23	12.28 ± 0.45
5	271.63 ± 6.92	12.30 ± 0.32	8.95 ± 0.10	25.59 ± 0.65	18.61 ± 0.37
25	258.31 ± 5.37	11.37 ± 0.25	8.27 ± 0.13	20.80 ± 0.71	15.13 ± 0.62
125	220.92 ± 6.15	9.72 ± 0.11	7.07 ± 0.09	15.26 ± 0.29	11.10 ± 0.18

Data are means ± SD.

## Data Availability

The data presented in the study are available on request from the corresponding author. The data are not publicly available due to the restriction policy of the coauthors’ affiliations.
